# 

**Published:** 1853-07-01

**Authors:** 


### OUR LIBRARY TABLE.

Our library table literally groans under the weight of a mass of books not directly connected with the speciality of this journal. We feel reluctant to lay ourselves open to a charge of discourtesy by omitting all notice of these valuable works, although we feel indisposed to trespass upon the province of our contemporaries, by giving any detailed analysis of them. With this apology, we hope our friends will kindly be satisfied with a brief reference to their respective labours in the paths of medical literature. *Dr. W. Stevens's "Observations on the Nature and Treatment of Asiatic Cholera,"* we have read with much pleasure, although we imagine he has rather overrated the value of the saline treatment advocated in the volume before us. The work should, however, be read by every medical man who is likely to be called upon to treat this dire disease. *Dr. Henry W. Fuller's "Rheumatism, Rheumatic Gout, and Sciatica, their Pathology, Symptoms, and Treatment,"* is one of the best practical works of modern times. It is evidently the production of a physician who has studied disease not only in books, but at the bedside of the patient. The work reminds us more of the late Dr. Gooch's elegant and classical work on the "Diseases of Females," than any work of recent date. The theoretical as well as practical remarks of Dr. Fuller upon rheumatism, gout, and sciatica, are extremely valuable, and show an intimate acquaintance with this class of affections. Dr. Fuller's position as assistant physician to St. George's Hospital, affords him a large field for observation and research. *Mr. W. F. Headland's prize essay "On the Action of Medicines, or the mode in which Therapeutic Agents introduced into the Stomach produce their peculiar effect upon the Animal Economy,"* &c., is a valuable contribution to the medical literature of the day. It is a work of profound research, and gives promise of great future eminence and excellence. Although exception may be taken to some of Mr. Headland's theories, we regard the book, as a whole, entitled to our warm approbation.

It has for many years been well known in the profession, that *Mr. Haynes Walton*, the distinguished surgeon to the Central Ophthalmic Hospital, was engaged in preparing for publication, an elaborate work on "*Operative Ophthalmic Surgery.*" His position, in connexion with the Ophthalmic Hospital, as well as his appointment as one of the surgeons to St. Mary's Hospital, afforded him a large field for the display of his great knowledge and eminent skill as a successful operator. His work has not disappointed us. It will for the future be a standard work in the profession. His style is simple and yet elegant; his instructions for performing the various difficult operations connected with the diseases of the eye, are lucid, and evidently proceed from the pen of a man well-accustomed to the use of the knife. Mr. Walton's views, as to the pathology and treatment of diseases of the eye in general, prove that he has been a close observer of nature. We feel much pleasure in recommending this work to the favourable consideration of those interested in this department of practical and operative surgery. *Dr. R. P. Cotton's Fothergillian prize essay "On the Nature, Symptoms, and Treatment of Consumption,"* has excited considerable notice in the profession. It is an excellent *resumé* of our knowledge of the phenomena and treatment of consumption, interspersed with much original observation. We trust Dr. Cotton will continue his labours in this interesting field of research. He has a mind admirably fitted for this enquiry. *Dr. Cockle* has published an interesting essay "*On the Poison of the Cobra Capello,*" which is worthy of the study of the toxicologist and physiologist. It is an elegantly written essay, showing great research and originality of observation.

---

### OXFORD UNIVERSITY.

At the Installation of the Right Hon. the Earl of Derby, as Chancellor of the University of Oxford, on the 9th of June, Dr. Forbes Winslow, the Editor of this Journal, had conferred upon him the honorary degree of Doctor in Civil Law.

